# The Gluteus Medius Vs. Thigh Muscles Strength Ratio and Their Relation to Electromyography Amplitude During a Farmer’s Walk Exercise

**DOI:** 10.1515/hukin-2015-0016

**Published:** 2015-04-07

**Authors:** Petr Stastny, Michal Lehnert, Amr Zaatar, Zdenek Svoboda, Zuzana Xaverova, Przemysław Pietraszewski

**Affiliations:** 1 Palacky University in Olomouc, Faculty of Physical Culture, Tr. Miru 115, post. 771 11 Olomouc, Czech Republic.; 2 Department of Theory and Practice of Sport; The Jerzy Kukuczka Academy of Physical Education in Katowice; Poland.

**Keywords:** isometric strength, loaded walking, electromyography, hip abduction, MVIC

## Abstract

The strength ratio between hamstrings and quadriceps (H/Q) is associated with knee injuries as well as hip abductor muscle (HAB) weakness. Sixteen resistance trained men (age, 32.5 ± 4.2 years) performed 5 s maximal isometric contractions at 75° of knee flexion/extension and 15° of hip abduction on a dynamometer. After this isometric test they performed a Farmer’s walk exercise to find out if the muscle strength ratio predicted the electromyography amplitude expressed as a percentage of maximum voluntary isometric contraction (%MVIC). The carried load represented a moderate intensity of 75% of the exercise six repetitions maximum (6RM). Electromyography data from the vastus medialis (VM), vastus lateralis (VL), biceps femoris (BF) and gluteus medius (Gmed) on each leg were collected during the procedure. The groups selected were participants with H/Q ≥ 0.5, HQ < 0.5, HAB/H ≥ 1, HAB/H < 1, HAB/Q ≥ 0.5 and HAB/Q < 0.5. One way ANOVA showed that Gmed activity was significantly greater in the group with HAB/H < 1 (42 ± 14 %MVIC) as compared to HAB/H ≥ 1 (26 ± 10 %MVIC) and HAB/Q < 0.5 (47 ± 19 %MVIC) compared to HAB/Q ≥ 0.5 (26 ± 12 %MVIC). The individuals with HAB/H < 1 were found to have greater activation of their Gmed during the Farmer’s walk exercise. Individuals with HAB/Q < 0.5 had greater activation of the Gmed. Gmed strength ratios predict the muscle involvement when a moderate amount of the external load is used. The Farmer’s walk is recommended as an exercise which can strengthen the gluteus medius, especially for individuals with a HAB/H ratio < 1 and HAB/Q < 0.5.

## Introduction

The strength ratio of hamstrings to quadriceps (H/Q) is one of the standard ways of evaluating knee stability (Holcomb et al., 2007; Kong and Burns, 2010; Lehnert et al., 2011) and to estimate knee injury potential or knee strength condition. Another approach used in injury prevention consists of evaluating the muscle activity of the vastus medialis (VM) and the vastus lateralis (VL) (Crossley et al., 2001) to estimate patella stability and patellofemoral pain (PFP) potential (Fagan and Delahunt, 2008; Crossley et al., 2001). Beyond this, there is evidence that hip instability influences knee stability as well as other muscle involvement around the knee area (Baffa et al., 2012; Felício et al., 2011). The H/Q ratio has already been used to estimate standards for the general population and injury prediction (Harbo et al., 2012; Danneskiold-Samsøe et al., 2009), as well as the VM/VL activity ratio (Irish et al., 2010; Van Tiggelen et al., 2009), but there are no standards for related muscle action in the hip area. Weakness in hip abductor muscles (HAB), as gluteus medius (Gmed), has been associated with both knee and hip instability (Hrysomallis, 2009; Carcia and Martin, 2007; Distefano et al., 2009), therefore, HAB strength can be used as a parameter to compare thigh muscle strength and activity. HAB strength has already been used for evaluating the ratio involving hip adductors (Tyler et al., 2001; Cichanowski et al., 2007), but there are no evaluations for the strength ratio between thigh muscles.

The HAB strength evaluation is traditionally performed by physiotherapists on athletes during convalescence and the tested subject performs abduction movements in the standard testing position. Such functional tests are limited in their quantitative outcome, therefore, muscle testing can be performed with a dynamometer in the same position (Arnold et al., 2010; Meyer et al., 2013; Bohannon, 1986; Andrews et al., 1996).

The aim of this study was to determine if H/Q, HAB/H and HAB/Q strength ratios could predict muscle activation during a Farmer’s walk exercise. The individual muscle strength ratios could have an impact on muscle activation in complex exercises such as squats or Farmers’ walks. For practical interpretation the H/Q ratio is standardized, therefore, it can be used as a baseline for HAB ratios. The findings of the present study can be used for the optimization of exercise selection and for detailed evaluation of the strength deficit of individual muscles.

## Material and Methods

### Experimental Approach to the Problem

The aim of this study was to prove if muscle strength ratios predicted muscle activation during complex exercises. Participants underwent the testing procedure in a cross-sectional study design. Assigning of participants to selected groups was based on the dynamometric results.

### Participants

The participants consisted of sixteen men involved in resistance training (age, 32.5 ± 4.2 years, body height, 184 ± 6.1 cm, body mass, 89 ± 9.2 kg) at a professional or amateur level in power-lifting (squat 1RM performance 170 ± 35 kg). They were divided into six groups according to their strength ratios (Table 1). All participants were involved in resistance-training programs of at least three sessions per week. They were free from any pathologies and injuries. Informed written consent was provided by each participant and the testing protocol was approved by the local Committee of Ethics in accordance with the ethical standards of the Declaration of Helsinki of 1983.

### Procedure

The warm-up procedure included five minutes of cycling and sets of 25 squats in five different foot positions followed by the EMG taping and maximal isometric performance evaluation. After the isometric tests, the participants were taped with 3D markers and asked to perform five 8 m walking trials with a progressively increased load. The load was increased up to six-repetition maximum (6RM), where the American Society of Exercise Physiology recommendations were followed (Brown and Weir, 2001). The rest intervals between the trials ranged from 30 to 60 s. The only measurement included in the statistical analysis was the one at 75% of 6RM. At this level, the Farmer’s walk looked like a six-step cycle where the subject was able to carry the load with shoulders retracted while keeping the trunk of his body in an upright position. The participants were instructed to perform the Farmer’s walk carrying dumbbells with shoulder retraction and their trunks held in the upright position, but were given no instruction for their lower limbs (preference being towards natural performance). The dumbbell handles were taped with sticky rubber to avoid slippage. The surface EMG was measured along with the 3D kinematics of walking to detect the knee and hip joint angles.

### Measures

#### Dynamometry

Isokinetic dynamometry was used to determine the unilateral muscle strength ratio and to normalize electromyography (EMG) amplitude using an “angle specific” method (Burden, 2010). The subjects performed 5 s of unilateral isometric contractions two times with a rest interval of 60 s on an IsoMed 2000 Dynamometer (D & R Ferstl GmbH, Hemau, Germany). To measure the H/Q ratio, isometric strength of the knee extensors and flexors was assessed in the standard sitting position with 75° knee flexion. The backrest of the dynamometer seat was set at a 75° angle and the angle of the hip joint was at 100°. Participants were held into position by pelvic and thigh belts while their lower limbs were tested. Adjustable pads and straps were placed at the shoulder-level and participants held the hand grips along the seat. The mechanical axis of the dynamometer was aligned with the participant’s knee axis of rotation utilizing the lateral femoral epicondyle as a bony reference. The distal shin pad of the dynamometer lever arm was attached with a strap of 2 cm proximal to the medial malleolus. HAB strength was measured twice in standard muscle testing positions for the Gmed with the measured lower extremity in a 15° hip abduction. The tested leg was fixed by straps to the dynamometer keeping the testing position of the leg constant. Before executing the maximal isometric contraction, the participant’s full range of motion was measured on the dynamometer. The axis of the dynamometer was then aligned with the greater trochanter on the femur and the dynamometer lever arm was fixed to the lateral thigh 1 cm above the patella on the tested limb.

#### Electromyography

Raw EMG signals of all muscles were collected bilaterally with the Noraxon Myosystem 1400A device (Noraxon; Scottsdale). The signals were recorded by eight leads with a frequency of 1000 Hz. Two bipolar surface electrodes (adhesive disposable electrode - Kendall) were placed with a 10 mm inter-electrode separation distance. The input impedance was greater than 10 MΩ at 100 Hz. The raw signal was transferred using an analogue connection to the 3D system (Vicon Data Log via MX box). The raw signal was operated simultaneously by the MyoResearch XP Master Version 1.03.05 program. EMG data were bandpass filtered (10–500 Hz) and smoothed using a root mean square followed by a window frame envelope with a time constant of 200/25 ms. The EMG signal was normalized to the maximum EMG value from isokinetic tests to the percentage of maximal voluntary isometric contraction (%MVIC). The mean amplitude expressed as %MVIC was chosen to describe the level of muscle activation. The electrodes for VM were placed over the distal third of the muscle belly and were oriented 55° to the vertical. The electrode for VL was placed over the muscle belly in the distal third and oriented 15° vertical (Gilleard et al., 1998). The Gmed was located by palpating the iliac crest and placing electrodes parallelly to the muscle fibres 33% of the distance between the iliac crest and greater trochanter (Bolgla and Uhl, 2005; 2007), similarly to those used by O’Sullivan et al. (2010) for the Gmed posterior part. The electrodes for the biceps femoral were placed over the distal third of the long head muscle belly. The ground electrode was placed over the tibia bone. The selected muscle model was derived from previous studies (Hertel et al., 2004; Felício et al., 2011; Baffa et al., 2012) with additional measurement of BF for observation of at least one hamstring muscle.

#### 3D kinematics

The kinematic data were recorded at a frequency of 200 Hz using a six-camera Vicon MX10 infra-red motion analysis system (Oxford Metrics, Oxford, UK). Cameras were spaced around the walking track with two force plates (Kistler Instrumente, Winterthur, Switzerland) in the middle. The force plates and EMG output were connected to the Vicon software via an MX box. Reflective markers measuring 19 mm in diameter were attached bilaterally on the subject’s skin overlying the following landmarks: the anterior superior iliac crest; the posterior superior iliac crest; the lateral thigh; the lateral femoral epicondyles; the tibias; the lateral malleolus; the heels; and the metatarsal head of the second toe. 3D kinematics was used to detect step cycles defined as heel-to-heel contact on the force plates with a sensitivity detection of 20N. When a non-periodical course of kinematic walking was observed during a subject’s Farmer’s walk, the attempt was excluded from the trials as “invalid”.

### Statistical Analyses

For statistical analysis, the participants’ results were divided into groups based on their H/Q, HAB/Hamstring (HAB/H) and HAB/quadriceps (HAB/Q) strength ratios to determine if the strength ratios predicted the electromyography amplitude of the VM, VL, BF and Gmed expressed as a %MVIC. The groups formed were participants with the results of H/Q ≥ 0.5 (H/Q 1), H/Q < 0.5 (H/Q 2), HAB/H ≥ 1 (HAB/H 1), HAB/H < 1 (HAB/H 2), HAB/Q ≥ 0.5 (HAB/Q 1) and HAB/Q < 0.5 (HAB/Q 2). The reliability across five trials of each loading condition was counted by an intraclass correlation coefficient (ICC) on a confidence interval CI = 0.95 to confirm if the EMG measurement was stable within a subject. In addition, Kruskal–Wallis tests were performed for each parameter between the groups to verify the normality of the distribution. A one-way analysis of variance (ANOVA) was used to compare if muscle activation had significant differences in measured loading conditions among the selected groups. The Tukey’s post hoc test was used to indicate significant differences. STATISTICA version 12 (StatSoft, Inc., Tulsa, OK, USA) software was used for the statistical analysis.

## Results

Reliability analyses across five trials for each subject resulted in a single-case intraclass correlation coefficient (ICCs) value ranging from 0.42 to 0.92 for VMO, BF, VL and Gmed, which is considered to be between a moderate to high level of reliability (Chandler and Brown, 2008; Chinn and Burney, 1987). The mean intraclass correlation coefficient (ICCm) ranged from 0.72 to 0.96 for all EMG values, which is considered to be between high and very high reliability.

**Figure 1:**
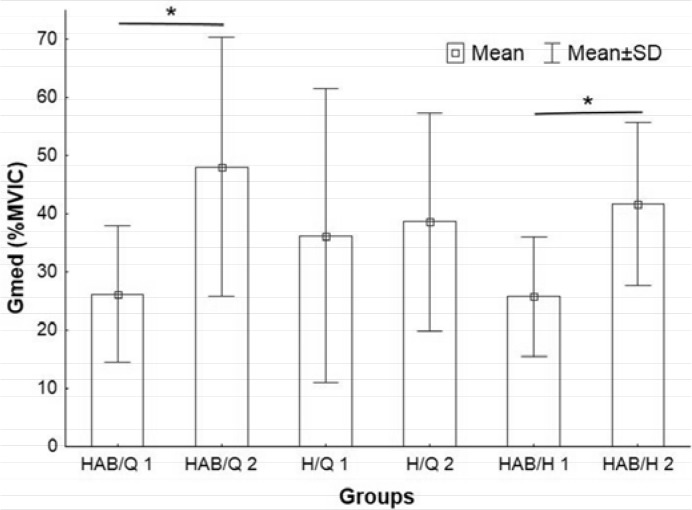
ANOVA result for Gmed Gmed = gluteus medius, %MVIC= percentage of maximal voluntary contraction. HAB = hip abductors. Q = quadriceps. HAB/Q 1 = group with HAB/Q ≥ 0.5. HAB/Q 2 = group with HAB/Q < 0.5, H/Q 1 = group with H/Q ≥ 0.5. H/Q 2 = group with HAB/Q < 0.5, HAB/H 1 = group with HAB/H ≥ 1. HAB/H 2 = group with HAB/H < 1, ^*^ significant difference by the Tukey range test.

The participants’ results were divided into six groups by their strength ratios shown in Table 1. All EMG parameters were tested by Kruskal–Wallis tests (Table 2) and one-way ANOVA; for the purpose of EMG analyses, trials when subjects performed the exercise with 75% of their 6RM were used. Significant differences were found in the Gmed values (F_5, 102_ = 4.5, p =.00097) between HAB/Q 1 and HAB/Q 2 and HAB/H 1 to HAB/H 2 (Figure 1).

The Gmed activity was significantly greater in the HAB/Q 2 (47 ± 18.7% MVIC) and HAB/H 2 (42 ± 14% MVIC) compared to the HAB/Q 1 (26 ± 12.1% MVIC) and HAB/H 1 (26 ± 10% MVIC), respectively. Other groups and muscle activity did not show significant differences (Table 2).

Individuals with a HAB/H < 1 have greater activation of the Gmed during a Farmer’s walk exercise. Individuals with a HAB/Q < 0.5 have greater activation of the Gmed. HAB strength ratios predict the muscle involvement for the Gmed during moderate intensity of 70% of 6RM during a Farmer’s walk exercise.

## Discussion

The finding that individuals with a HAB/H < 1 have greater activation of the Gmed during a Farmer’s walk exercise with 75% of 6RM suggests that, in this case, the weaker muscle groups have to work in order to achieve a higher level of activity. Thus, the group with stronger HABs than hamstrings shows more equal activity increases than groups with weaker HABs. Weaker HABs can be assumed as muscle imbalances even in athletes who follow resistance training without any pathology issues, which is in agreement with other studies (McCurdy et al., 2010; Reiman et al., 2012). Previous studies have also found that the HAB/hip adductor (HAD) strength ratio should be about 0.95 (Tyler et al., 2001), when a HAB/HAD ratio of 0.78 was found in injured athletes. The results of this study suggest that a HAB/H ratio with a separation border of 1 influences the level of muscle bioelectrical activity of the Gmed.

Individuals with a HAB/Q < 0.5 have greater activation of the Gmed. This finding supports the idea that HAB weakness results in changes in Gmed muscle activity. Therefore, the hypothetical HAB/Q ratio of 0.5 cannot be assumed as the main separation border for the prediction of the Gmed bioelectrical activity because no similar findings have been reported in previous studies. To make such a statement, more support findings are necessary. The H/Q strength ratio showed no significant results, likely due to the type of exercise chosen – an exercise that did not include the use of the posterior muscle chain.

One limitation of our study is that the EMG is specific to the selected load (75% of 6RM). Additionally, muscle activity can vary between individuals due to genetics profile (Petr et al., 2014) or the type of exercise (Čoh 2011 and Žvan). This study used a moderate level of exercise intensity to avoid measuring of muscle bioelectrical activity due to kinematic changes. In addition, this type of moderate-intensity exercise is used in the early phases of resistance training amongst both trained and untrained subjects when strength endurance and exercise technique is the aim (Siff, 2003). Thus, the results of this study should be used with regard to early phases of the training cycle. With these points in mind, it is important to determine whether muscle’s activation is similar when the load of the external resistance is increased or if a subject’s strength levels change over time. Another limitation is the number of measured muscles, as only four muscles were measured bilaterally. For example gluteus maximus, which plays an important role in the walking pattern (Pandy et al., 2010) was not included according to the selected muscle model.

The subjects included in this research group were specifically recruited because of their resistance-training level. This specific population has previously been reported as having higher MVICs results (Maeo et al., 2013) when resistance training was performed twice a week which led to significant increases in the maximal voluntary activation of VL and VM muscles during both isometric and concentric knee extension actions (Häkkinen et al., 2001) and RMS values (Dasteridis et al., 2012). However, the measured values observed for VM, VL and BF (Table 2) are considered as low (Burden, 2010). This might be the cause of short muscle length change (Jönhagen et al., 2009) of these muscles during the Farmer’s walk and due to the low amount of the used load (75% of 6RM) for resistance trained subjects. On the other hand, Gmed values (Table 2) above 41% of MVIC are considered as a high level of activation and between 21 to 40% as a moderate level of activation (Reiman et al., 2012).

An important part of the measurement was estimation of the body position for testing HAB strength. Different body positions and testing procedures were previously reported as of good reliability (Bohannon, 1986; Reiman et al., 2013). Due to the similarity in testing trained subjects, this study used a similar position to the one applied in a recent study (Burnet and Pidcoe, 2009).

The main outcome of the present study is that the HAB and thigh muscles strength ratios predict the muscle involvement during a Farmer’s walk exercise. This leads us to support the idea that the HAB ratio may be beneficial for determining the level of muscle activity during other exercises other than the Farmer’s walk. For example, Gmed activity has been measured in body-weight conditions during forward lunges and single limb deadlift (Boren et al., 2011; Distefano et al., 2009) without estimating muscle strength. The Farmer’s walk is recommended as an exercise in order to strengthen the Gmed, especially for individuals with a HAB/H ratio < 1 and a HAB/Q < 0.5.

## Conclusion

The HAB to thigh muscle strength ratio can be used to estimate the appropriate strength or weakness of the Gmed. The Farmer’s walk exercise should be used as an exercise to strengthen the Gmed, especially in groups with a HAB weakness.

## Figures and Tables

**Table 1:** Description of research groups by dynamometry unilateral isometric test (n = 32) H/Q 1, hamstring/quadriceps group 1; H/Q 2, hamstring/quadriceps group 2; HAB/H 1, hip abductor/hamstring group 1; HAB/H 2, hip abductor/hamstring group 2; HAB/Q 1, hip abductor/quadriceps group 1; HAB/Q 2, hip abductor/quadriceps group 2; ^*^ muscle ratio of the group listed in the first column;

	Knee Flexion 75° Mean ± SD (N·m^−1^)	Knee extension 75° Mean ± SD (N·m^−1^)	Hip abduction 15° Mean ± SD (N·m^−1^)	^*^Ratio Mean ± SD

H/Q 1 (n= 13)	135 ± 22	289 ± 55	166 ± 30	0.58 ± 0.06
H/Q 2 (n= 19)	157 ± 16	324 ± 60	131 ± 14	0.41 ± 0.05
HAB/Q 1 (n= 17)	152 ± 21	276 ± 35	144 ± 29	0.55 ± 0.06
HAB/Q 2 (n= 15)	137 ± 22	327 ± 65	158 ± 29	0.43 ± 0.04
HAB/H 1 (n= 17)	133 ± 19	294 ± 55	167 ± 30	1.26 ± 0.13
HAB/H 2 (n= 15)	161 ± 16	316 ± 62	130 ± 13	0.82 ± 0.11

**Table 2:** Muscle activation at 75% of 6RM by selected groups (n = 32) %MVIC, percentage of maximal voluntary isometric contraction; VMO, vastus medialis; VL, vastus lateralis; BF, biceps femoris; Gmed, gluteus medius; H/Q 1, hamstring/quadriceps group 1; H/Q 2, hamstring/quadriceps group 2; HAB/H 1, hip abductor/hamstring group 1; HAB/H 2, hip abductor/hamstring group 2; HAB/Q 1, hip abductor/quadriceps group 1; HAB/Q 2, hip abductor/quadriceps group 2.

	VM Mean ± SD (%MVIC)	VL Mean ± SD (%MVIC)	BF Mean ± SD (%MVIC)	Gmed Mean ± SD (%MVIC)
H/Q 1 (n= 13)	16 ± 4.5	19 ± 8.5	14 ± 7.5	36 ± 25.2
H/Q 2 (n= 19)	16 ± 7.7	14 ± 4.4	10 ± 6.2	39 ± 18.7
HAB/Q 1 (n= 17)	16 ± 6.8	16 ± 8.3	13 ± 6.6	26 ± 12.1
HAB /Q 2 (n= 15)	16 ± 7.7	14 ± 4.4	10 ± 6.2	47 ± 18.7
HAB /H 1 (n= 17)	17 ± 8.9	17 ± 7.7	15 ± 7.5	26 ± 10
HAB /H 2 (n= 15)	15 ± 3.0	15 ± 5.0	8 ± 3.5	42 ± 14.0
Kruskal–Wallis (p)	0.99	0.47	0.12	0.06
